# RECOGNIZING CAREGIVING NEEDS OF CHINESE AMERICAN OLDER ADULTS AND EXPLORING CHATBOTS UTILIZATION

**DOI:** 10.1093/geroni/igae098.0699

**Published:** 2024-12-31

**Authors:** Yanjing Liang, Serena Jinchen Xie, Jingyi Li, Jingjing Xu, Weichao Yuwen

**Affiliations:** University of Washington, Seattle, Washington, United States; University of Washington Medicine, Seattle, Washington, United States; University of Washington Tacoma, Tacoma, Washington, United States; The First Affiliated Hospital of Nanjing Medical University, Nanjing, Jiangsu, China (People’s Republic); University of Washington Tacoma, Tacoma, Washington, United States

## Abstract

Asian Americans encounter unique challenges in caring for older adult family members due to language barriers, cultural preferences, and difficulty in accessing healthcare resources. Family caregivers of Chinese-speaking older adults in the U.S. reported high levels of emotional stress more often compared to English-speaking caregivers. Digital health tools such as chatbots have the potential to offer affordable and accessible support. This qualitative study examined data from 12 in-depth interviews of families caring for Chinese American older adults and provided insights into caregiving challenges, self-care practices, cultural values, and perceptions of using chatbots for support. Participants faced challenges, including barriers to accessing resources due to language, limited understanding of social support, and stigma. The high-power distance of Chinese culture (Hofstede cultural value theory) alongside ‘familism’ and ‘filial piety’ exacerbated challenges in intergenerational communication. We discovered that the psychological aspect of self-care among Chinese American caregivers is often undervalued, influenced by the culture of self-restraint and endurance. Participants anticipated chatbots providing lifelike support and comprehensive caregiving resources by integrating functions such as health information guides. While caregivers appreciated chatbots as a judgment-free channel for seeking help, they had reservations about chatbots’ capacity to comprehend users’ emotions and embody cultural nuances such as sayings and references, which would make chatbots relatable and encourage engagement. The effectiveness of chatbots may be limited by constraints in empathy and cultural sensitivity. The findings reveal both the promise and challenges of developing culturally sensitive chatbots to address emotional needs and offer comprehensive support.

